# The Pharmacological Treatment of Neuropathic Pain in Children

**DOI:** 10.2174/1570159X21666230804110858

**Published:** 2023-08-04

**Authors:** Lisa M. Einhorn, Jonathan Hudon, Pablo Ingelmo

**Affiliations:** 1 Department of Anesthesiology, Pediatric Division, Duke University School of Medicine, Durham, North Carolina, United States;; 2 Division of Secondary Care, Department of Family Medicine, McGill University Health Centre, Montreal, Qc, Canada;; 3 Palliative Care Division, Jewish General Hospital, Montreal, Qc, Canada;; 4 Alan Edwards Pain Management Unit, Montreal General Hospital, McGill University Health Center, Montreal, Qc, Canada;; 5 Alan Edwards Centre for Pain Research, McGill University, Montreal, Canada;; 6 Edwards Family Interdisciplinary Centre for Complex Pain, Montreal Children’s Hospital, McGill University Health Center, Montreal, Canada;; 7 Research Institute of the McGill University Health Center, Montreal, Canada;; 8 Department of Pediatric Anesthesia, Montreal Children’s Hospital, McGill University Health Center, Montréal, QC, Canada

**Keywords:** Neuropathic pain, neuralgia, pediatric, children, pharmacology, analgesia

## Abstract

The International Association for the Study of Pain (IASP) defines neuropathic pain as pain caused by a lesion or disease of the somatosensory nervous system. It is characterized as a clinical condition in which diagnostic studies reveal an underlying cause of an abnormality in the peripheral or central nervous system. Many common causes of neuropathic pain in adults are rare in children. The purpose of this focused narrative review is, to 1) provide an overview of neuropathic pain in children, 2) highlight unique considerations related to the diagnosis and mechanisms of neuropathic pain in children, and 3) perform a comprehensive analysis of the pharmacological treatments available. We emphasize that data for routine use of pharmacological agents in children with neuropathic pain are largely inferred from adult literature with little research performed on pediatric populations, yet have clear evidence of harms to pediatric patients. Based on these findings, we propose risk mitigation strategies such as utilizing topical treatments whenever possible, assessing pain phenotyping to guide drug class choice, and considering pharmaceuticals in the broader context of the multidisciplinary treatment of pediatric pain. Furthermore, we highlight important directions for future research on pediatric neuropathic pain treatment.

## 1 INTRODUCTION

In 2011, the International Association for the Study of Pain (IASP) published a new definition of neuropathic pain. In this definition, neuropathic pain occurs as a direct consequence of a diagnosis, proven lesion or disease of the somatosensory system [1]. The goal of this narrower definition was to provide a mechanistically driven approach to studying neuropathic pain syndromes through consistent clinical and pharmacological research [2]. However, this more restrictive definition excluded patients suffering from conditions with centrally maintained pain of unexplained etiologies or unproven causes. To address the gap in research and clinical terminology, in 2017, the IASP introduced the term “nociplastic pain” which is defined as “pain that arises from altered nociception despite no clear evidence of actual or threatened tissue damage causing the activation of peripheral nociceptors or evidence for disease or lesion of the somatosensory system causing the pain”. However, the definition of nociplastic pain remains a controversial topic in the literature [3], and methods to discriminate between pain mechanism categories continue to be refined [4].

The prevalence of neuropathic pain in children is not known [5, 6]. It has been well established that the most common causes of neuropathic pain in adults, including painful diabetic neuropathy, postherpetic neuralgia, trigeminal neuralgia, and cerebrovascular accident, are rarely, if ever, seen in pediatric practice [7, 8]. While pain in children is common, a somatic etiology can only be identified in 10-30% of children with unexplained pain [9]. Centrally maintained pains without observable pathology in children include chronic widespread pain, primary headache or orofacial pain, primary visceral pain, complex regional pain syndrome, and primary musculoskeletal pain [10]. In the absence of nervous system injury, these clinical disorders do not meet the IASP definition of neuropathic pain. Therefore, for the purpose of this review, the focus will be on the management of neuropathic pain for specific disease processes in children as defined by the IASP. A list of these causes is found in Table **1**.

## 2 CLINICAL MANIFESTATIONS

Patients with neuropathic pain typically complain of ongoing spontaneous pain most often described as burning, shooting, pins and needles, squeezing, throbbing, or freezing [11]. There may also be intermittent electric-shock paroxysms either alone or in addition to ongoing pain. Because of the nervous system lesion, other abnormal sensations including dysesthesias (an unpleasant abnormal sensation) and paresthesias (an abnormal sensation that is not unpleasant) may occur either spontaneously or evoked [1]. Evoked type pain (touch-evoked, cold-evoked) rarely occurs as the only manifestation. On examination, both allodynia (pain due to a stimulus that does not normally provoke pain) and hyperalgesia (increased pain from a stimulus that normally provokes pain) may be present to tactile or thermal stimulation [1]. Other associated manifestations include 1) hyperpathia, an often explosive pain response characterized by an abnormal reaction to a repetitive stimulus at increasing thresholds [1]; 2) aftersensations, pain which continues after cessation of the stimulus [12]; and 3) referred sensations, an abnormal response to stimulation in which paresthesias, pressure, and changes in temperature or pain intensity were referred to various non-stimulated areas [13].

Similar sensations have been reported by children ages 6 years and older [14, 15]. Observer based tools of pain behavior have been validated for acute and chronic pain in infants and preverbal or cognitively delayed children [16, 17] but distinguishing neuropathic pain from other pain states in this population is challenging. Strong clinical suspicion due to observed abnormal sensation, such as lack of awareness of a noxious stimulus, combined with a confirmed lesion or disease process in the somatosensory system may allow for the differentiation of neuropathic pain in young or cognitively delayed children.

## 3 DIAGNOSIS

**Fig. (1):**
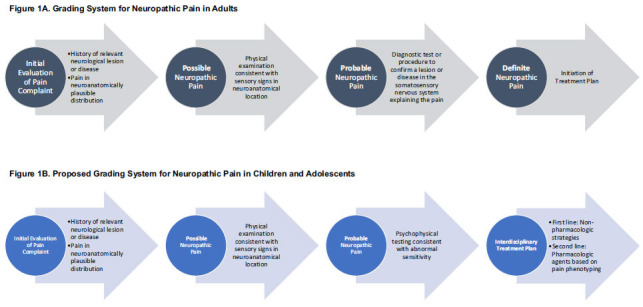
(**A**) Grading system for neuropathic pain in adults adapted from Finnerup *et al*. [23] and (**B**) Proposed grading system for neuropathic pain in children and adolescents. Key differences between the Fig. (**1A** and **1B**) begin at the third steps in the respective algorithms. In Figure **1A**, “definite” neuropathic pain means “probable neuropathic pain with confirmatory tests.” However, objective diagnostic testing is limited in children compared to adults. In the absence of genetic tests or neuroimaging to confirm a medical diagnosis, psychophysical testing, while subject to patient participation, can inform an interdisciplinary treatment plan.

Neuropathic pain cannot be diagnosed by a singular biomarker, specific test, or isolated exam finding. Certain clinical conditions such as pain associated with postherpetic neuralgia and diabetic neuropathy in adults and Fabry disease in children have a well-defined underlying cause. However, common conditions (*e.g*. low back pain) have been described as “mixed pain” [18], characterized by a complex overlap of both nociceptive and neuropathic pain, which can present challenges for diagnosis [19]. A variety of pain scales and clinical tools have been developed for assessment and diagnosis of neuropathic pain in adults (*e.g*. Leeds Assessment of Neuropathic Syndomes and Signs (LANSS) [20], Douleur Neuropathique 4 questionnaire (DN4) [21], Neuropathic Pain Questionnaire (NPQ) [22]). However, no study has shown that these applications are valid, reliable, and generalizable to a range of conditions, with differing severity, over time [19, 23-25]. For this reason, a hierarchical system has been developed [24], and subsequently revised [23], to grade neuropathic pain into three levels: possible, probable, and definite (Fig. **1A**). The different levels are based on, 1) the clinical history of a relevant disease process and reported pain distribution which is anatomically possible; 2) physical exam with sensory signs consistent with the pain distribution; and 3) diagnostic testing to confirm the presence of the lesion or disease explaining the pain. Other types of pain should be considered and determined to be an unlikely cause of symptoms.

While these diagnostic guidelines were developed for adults, it may be reasonable to extrapolate them to older children and adolescents, who 1) can self-report pain histories, descriptors, location, and intensity with reliability, 2) have a physical exam with sensory changes that may overlap, be within, or extend beyond the pain distribution, and 3) can undergo psychophysical testing to quantify sensitivity thresholds (Fig. **1B**). Quantitative sensory testing (QST) is a well-developed comprehensive examination of somatosensory function of all primary afferents [26]. Large fiber function (Aβ) is tested by mechanical detection thresholds to von Frey hairs and vibration. Nociceptive and non-nociceptive small fiber (Aδ, C) function and the spinothalamic pathways are tested by cold and warm detection, mechanical pain, and heat pain thresholds [26]. Testing is tolerated and feasible in children as young as 9 years old and is advantageous in the pediatric population because it is both non-invasive and standardized with age normative guidelines [27]. Conditioned pain modulation (CPM) is a centrally processed measure which evaluates endogenous descending pain inhibitory control [28]. Recent work using QST/CPM paradigm to develop phenotyping sensory profiles in children will be highlighted further in a later section of this review [29, 30].

## 4 BRIEF OVERVIEW OF PRE-CLINICAL MODELS INVOLVING EARLY LIFE NERVE INJURY

Our current understanding of the complex mechanisms associated with neuropathic pain is beyond the scope of this manuscript; however, several recent comprehensive reviews of the advances and insights gained from primary studies in rodent models have been published [19, 31-33]. Briefly, there are an array of neuropathic pain models that have been developed in animals to simulate behavioral responses to painful peripheral stimuli [34]. Commonly employed models for physical damage to peripheral nerves include the Spared Nerve Injury (SNI) model, involving the ligation and transection of the common peroneal and tibial branches of the sciatic nerve [35], and the Chronic Constriction Injury (CCI) model, resulting in inflammation induced nerve damage [36]. The consequence of these injuries in adult animals is the rapid onset of neuropathic pain, characterized by allodynia and pain hypersensitivity from the partially denervated regions [37].

This section will highlight the unique mechanistic findings associated with early life nerve injuries in animal models and their potential relationship to clinical findings in human infants and young children. Unlike adult animals, it has been well established that neuropathic pain is absent or transient in neonatal rat models. Multiple studies using SNI and CCI models in rat pups with nerve injuries prior to post-natal day 21 have shown that these animals display minimal to no allodynia and only transiently observed pain behaviors [38-40]. The absence of neuropathic pain in early life nerve injury has been attributed to the weak glial response in the dorsal horn of the spinal cord. Unlike adults, who develop a rapid onset proinflammatory response leading to sensitization of neurons in the dorsal horn, SNI in the neonatal period causes little or no increase in the expression of either microglia or astrocyte markers and no increase in the expression of pro-inflammatory mediators [41]. Neuropathic pain is similarly not reported or observed following peripheral nerve injuries in neonatal and young children [8, 42, 43].

However, early life nerve injuries in SNI rodent models have been shown to result in a delayed onset mechanical hypersensitivity emerging in adolescence due to neuroimmune activations and N-methyl-D-aspartate (NMDA) dependent central sensitization [44, 45]. These findings are consistent with increasing clinical evidence that repeated noxious stimulation as a result of tissue injury or excess nociceptive input in human infancy may result in prolonged structural and functional alterations in pain pathways lasting till later childhood, adolescent, and adult life [35, 44, 46-49]. It has been shown that the expression of proinflammatory markers (IBA1, BDNF, TNFα) increases with age following peripheral nerve injury in infancy. This proinflammatory response corresponds to a delayed onset of hypersensitivity and an increase in spontaneous and evoked activity in dorsal horn neurons, similar to that seen after adult nerve injury [45]. This timing is also consistent with age maturation of toll like receptor (TLR) inducible cytokine and chemokine release from microglia, which is low in the neonatal period but rises considerably towards adolescence [50]. Thus, the age at which nerve damage occurs is an important predictor of the development of neuropathic pain both in the short-term and long-term [43]. Older children and adolescents are more likely to develop a response similar to adults, but infants and young children may have a significant delay between nerve injury and the development of neuropathic pain. This is supported by studies which have shown that children with early amputations typically develop phantom pain after a mean of 7 years [51]. Furthermore, it has been hypothesized that older children with complex pain syndromes with little or no measurable disease at the time of presentation (“medically unexplained” or centrally maintained pain) may actually represent late development or “unmasking” of neuropathic pain from an early life injury [41].

## 5 TREATMENT OF CHRONIC PAIN IN CHILDREN

Few prospective controlled studies have been performed for the treatment of chronic pain in children. Interventions and treatment strategies are almost exclusively prescribed based on adult data. In 2017, three Cochrane reviews were published on the use of antiepileptics, antidepressants, and opioids for chronic non-cancer pain in children and adolescents [52-54]. Due to the lack of eligible studies and low quality of evidence, the authors found no evidence to support or refute the use of these medications in the pediatric population. These systematic reviews and others were summarized in a comprehensive overview published in 2019 [55], which concluded “we know little about the safety and efficacy of pharmacological medicines for children and adolescents with chronic pain, despite their common use.” The World Health Organization similarly published guidelines for the management of chronic pain in children in 2012, revised in 2020 [56], which reported “significant research gaps related to the effectiveness and safety of pharmacological interventions for chronic pain in children.”

Research gaps specifically in the management of neuropathic pain in children are even more striking. We will review the evidence available for the pharmacological treatment of neuropathic pain in children in the subsections below.

## 6 NEUROPATHIC PAIN TREATMENTS

### 6.1 Antiepileptics

#### 6.1.1 Gabapentinoids

Gabapentinoids are derived by the addition of a cyclohexyl group to the backbone of the gamma-aminobutyric acid (GABA). The mechanism of action is the pre-synaptic inhibition of voltage gated calcium channels by selective antagonism of the α2-δ subunit. This high-affinity binding site in brain membranes results in a reduction in the release of excitatory neurotransmitters such as glutamate [57]. In rodent models of neuropathic pain, gabapentin has shown to have anti-allodynic effects and an attenuation of neuropathic pain behaviors [58].

Neither the European Medicines Agency (EMA) nor the Food and Drug Administration (FDA) have approved the use of gabapentinoids for pain in pediatrics. Despite this, gabapentin is used off-label as an analgesic treatment for a wide range of diagnoses in children. A recent study published prescribing patterns of gabapentinoids over a 7-year period at a tertiary care Children’s Hospital [59]. The authors reported that the number of pediatric patients receiving prescriptions for gabapentin and pregabalin increased by 1.4 and 1.3-fold, respectively, between 2013-2019 with less than 20% for patients with a previous epilepsy diagnosis. Indications for prescriptions ranged from widespread musculoskeletal pain and neuropathic pain (most common) to abdominal/pelvic pain and headache (less common).

While the use of gabapentinoids is increasing in pediatric patients, there is minimal data to support their analgesic effect, even within the context of known neuropathic pain conditions. Publications in the form of individual case reports and small series have generally showed favorable results in the management of severe peripheral injury [60, 61], Fabry disease [62], phantom limb pain [63], and burn associated neuropathic pain [64]. There is a single center, open label, industry supported study (n = 30) of pregabalin in pediatric oncology patients which showed marked improvement in neuropathic pain symptoms in the majority of patients [65]. In 2011, a larger retrospective study (n = 498) was published in pediatric patients with acute lymphoblastic leukemia with chemotherapy induced peripheral neuropathy (CIPN) [66]. The study was limited by inconsistent data collection, lack of standardization assessment tools, and absence of data on medication adherence in the outpatient setting, and therefore, could not draw substantial conclusions. In 2020, these authors followed up this retrospective study with a double blind prospective randomized controlled trial (RCT) in vincristine treated pediatric cancer patients. 50 patients ages 1-18 years were enrolled to receive either opioids with placebo or opioids with 20 mg/kg/day of gabapentin for 21 days. Results of this well-designed trial showed no statistically significant difference in opioid consumption between groups, suggesting that gabapentin did not provide additional analgesic benefit [67]. Despite these negative study results, many published algorithms for the management of neuropathic pain suggest gabapentinoids as a first line pharmacologic treatment, either prophylactically or rescue medication in pediatric cancer patients [68-70].

There is currently only one RCT evaluating the treatment effect of gabapentin *versus* amitriptyline in pediatric patients with non-cancer neuropathic pain [71]. This study included 34 patients ages 7-18 years with either a diagnosis of CRPS type I (n = 20) or a neuropathic pain condition (n = 14) who were then randomized to gabapentin 300 mg three times per day or amitriptyline 10 mg at bedtime with placebo to maintain blinding for 6 weeks. There was no control arm included. The primary outcome was pain intensity and secondary outcomes included sleep quality and adverse events. The authors defined the minimally important difference (MID) of pain reduction as a change of pain intensity of 1 point on the Coloured Analogue Scale, which is assessed in 0.25-point scoring increments (range 0-10). At the end of the 6-week trial, both drugs were similarly effective in reducing pain levels (MID ≥ 1) among patients (46% in the amitriptyline group *versus* 60% gabapentin group, *p* = 0.71). There were no statistically significant differences in the treatment effects between groups in pain intensity, sleep scores, or adverse events. The authors acknowledge significant limitations of this study including lack of a placebo arm which was due to both lack of resources and ethical concerns of treating children with chronic pain with an inactive agent. In addition, there were numerous etiologies for neuropathic pain and the inclusion of CRPS type I, which is not a neuropathic pain syndrome as defined by the IASP, resulted in a heterogenous population in an already small, and potentially underpowered, trial. Nonetheless, despite its major limitations, this study published in 2016, remains the only prospective RCT evaluating the effect of gabapentin on non-cancer neuropathic pain in children.

With little evidence to support its analgesic benefit in children, the initiation of gabapentinoid therapy must be carefully weighed against its known side effects. Adverse effects include sedation, weight gain, edema, blurry vision, and dizziness. Gabapentinoids have been associated with more concerning changes in behaviors in adolescents, including an increased risk of suicidal ideation, unintentional overdose, aggression, and head/bodily injuries [72]. The abuse potential of gabapentinoids has been well-documented as sought-after agents to potentiate the effect of opioids [73]. Suicide risk-mitigation practices and substance misuse screening should be routinely employed.

#### 6.1.2 Perampanel

Perampanel is an antiepileptic medication approved to treat both partial and generalized seizure disorders in patients older than 12 years of age [74]. It is a non-competitive inhibitor of the Alpha-amino-3-hydroxyl-5-methyl-4-isoxazole-propionate (AMPA) receptor which meditates excitatory transmissions and glutamine receptors. In rodent models, perampanel was found to reduce pain perception in all behavioral tests as well as mechanical allodynia and hyperalgesia induced by the CCI model of neuropathic pain [75-77].

There is a single case report of an adult patient with CRPS type I who had a resolution of chronic lower extremity pain with the addition of perampanel to her regimen [78]. There are currently no reports of off label use for neuropathic pain in pediatric patients. Of note, the drug carries a black box warning due to the potential for serious psychiatric and behavioral changes including suicidal and homicidal ideation, psychosis, aggression, and delirium.

### 6.2 Antidepressants

Antidepressants are routinely used off-label for pain management at lower doses than what is required for the treatment of depression [53]. There are multiple classes of antidepressants that have been investigated for their potential analgesic benefits including tricyclic antidepressants (TCAs), selective serotonin reuptake inhibitors (SSRIs), and serotonin and norepinephrine reuptake inhibitors (SNRIs), and these are often incorporated into adult Clinical Practice Guidelines (CPGs) for neuropathic pain [79-82]. Bupropion, a norepinephrine dopamine reuptake inhibitor (NDRI), is an atypical antidepressant which has also been used to treat neuropathic pain in adults. All classes of antidepressants discussed below carry FDA black box and EMU safety warnings that with the initiation of treatment children and adolescents with major depressive disorder (MDD) may experience an increase in suicidal ideation.

#### 6.2.1 Tricyclic Antidepressants

As a class, TCAs act on multiple receptors. They inhibit serotonergic and noradrenergic reuptake, possess anti-inflammatory activity *via* prostaglandin and TNF-alpha modulation, and exhibit N-Methyl-D-aspartic acid (NMDA) antagonism, sodium and calcium channel inhibition, and kappa and delta opioid receptor agonism [83]. TCAs are typically separated into 2 categories based on their chemical structure, tertiary or secondary amines. Historically, tertiary amines (amitriptyline, doxepin, imipramine) have been more frequently prescribed due to a more balanced chemical profile and inhibition of both serotonin and norepinephrine equally [84]. The secondary amine nortriptyline has less serotonin reuptake inhibition but is associated with less sedation and fewer anti-cholinergic side effects.

TCAs are not approved by the FDA nor the EMA for chronic pain management in children. In practice, TCAs are most frequently used off-label for centrally maintained pain without supportive evidence. A multicenter, randomized, placebo-controlled trial of amitriptyline in children with unexplained gastrointestinal disorders found no significant differences between amitriptyline and placebo after 4 weeks of treatment [85]. Subsequently, a recent systemic review on the use of antidepressants for children with functional abdominal pain reports the inability to develop meaningful conclusions on the effectiveness of these medications in this population [86].

In children with diagnosed neuropathic pain syndromes, TCAs are often included in a multimodal regimen of pain management strategies [87, 88]. However, apart from the RCT discussed in the previous section (6.1) [71], there are no other pediatric trials evaluating the efficacy of TCAs for known neuropathic pain conditions in children. In adult literature, multiple Cochrane reviews found no high-level evidence to support or refute the use of TCAs for routine management of neuropathic pain [89-91]. Despite these results, TCAs are recommended for adults as a first line treatment for neuropathic pain [79-82]. Side effects include dry mouth, constipation, urinary retention, blurred vision, and arrhythmias at higher doses.

#### 6.2.2 Selective Serotonin Reuptake Inhibitors

There are no studies evaluating SSRIs in pediatric patients with neuropathic pain. In adult literature, SSRIs provide limited and inconsistent analgesic benefit for adult neuropathic pain conditions [92, 93] and are generally considered fourth line treatments in adult CPGs [82].

#### 6.2.3 Serotonin and Norepinephrine Reuptake Inhibitors

Unlike SSRIs, SNRIs are balanced and increase both serotonergic and noradrenergic neurotransmission. SNRIs are multi-mechanistic like TCAs but may be easier to use in clinical practice due to their reduced anticholinergic and antihistaminergic effects, theoretically resulting in fewer side effects [94].

In pediatric patients, duloxetine is approved for use in children ages 7-17 years for generalized anxiety disorder and MDD but has no approved indications for pain management. Despite its success in analgesia for chemotherapy-induced peripheral neuropathy (CIPN) in adults [95, 96], duloxetine has not been suggested in algorithms to manage neuropathic pain associated with chemotherapeutic agents in children [68, 69]. It has been used off-label in 5 case reports with a cumulative total of 11 pediatric patients with both chronic pain and comorbid psychiatric conditions [97-101]. In the largest of these series (n = 5) [100], each case resulted in discontinuation of duloxetine because of adverse events (nausea) and/or perceived lack of efficacy. One case report noted the development of a rare side effect, the syndrome of inappropriate antidiuretic hormone (SIADH) in a 10-year-old child with acute lymphoblastic leukemia on duloxetine to manage CIPN and comorbid anxiety [101]. There are currently no published prospective trials evaluating the use of duloxetine for neuropathic pain in children.

#### 6.2.4 Norepinephrine Dopamine Reuptake Inhibitors

Bupropion is an NDRI widely used for the acute treatment of attention-deficit/hyperactivity disorder and other disorders in children. There are no studies evaluating NDRIs in pediatric patients with neuropathic pain. In the adult literature, there is an open label study in 22 patients with a diagnosis of neuropathic pain which showed that approximately 70% had improvement of their pain over an 8-week period [102]. The same authors then published a double-blind randomized trial in 41 patients with similar results [103].

### 6.3 Opioids

Opioid pharmacology is complex, and our understanding of opioid receptor function continues to evolve [104]. In brief, opioids act on G-protein coupled receptors (mu, delta, kappa opioid receptors) located on the pre and post synaptic sites in the spinal cord, medulla, thalamus, periaqueductal gray, nucleus raphe, and cortex. Opioids alter the synaptic potentials affecting the transmission of neuropeptides involved in pain pathways in the peripheral and central nervous system [105].

There are no prospective randomized trials to support the routine use of opioids for neuropathic pain in children [55]. In adult literature, opioids have been shown to be more effective than placebo for the treatment of neuropathic pain; however, there is significant heterogeneity in the studied conditions limiting these results [106]. A Cochrane review published in 2013 analyzed 31 trials using 10 different opioids and demonstrated moderate pain relief (defined as at least 30% improvement) in 57% of study participants receiving an opioid *versus* 34% receiving a placebo (NNTB 4, 95% CI 2.7 to 7.7). 47% of study participants receiving an opioid reported substantial pain relief (defined as 50% improvement) *versus* 30% of patients receiving a placebo (NNTB 5.9, 95% CI 3.0 to 50.0) [106]. CPGs for adults differ in their recommendations for initiating opioids for neuropathic pain management, some suggesting they should be considered as second line agents and others third to fourth line [79, 80, 82].

#### 6.3.1 Centrally Acting Opioids

Certain opioids have unique mechanisms of action. Tramadol has a weak affinity for the mu opioid receptor but also acts centrally by inhibiting norepinephrine and serotonin reuptake in the spinal cord. It is thought this range of properties and additional central action may contribute to its analgesic effect for neuropathic pain [107]. Tramadol is recommended as a second line agent in multiple adult CPGs [79, 80]. In 2017, the FDA released a statement that tramadol is contraindicated in children under 12 due to metabolic variation of the cytochrome P450 enzyme CYP2D6 in the general population [108]. Ultra-rapid metabolism of tramadol leads to a rapid generation of the active metabolite, which has resulted in respiratory depression and deaths in children less than 12 years. There is an FDA black box warning for its use in children ages 12-17 years.

Tapentadol is another atypical opioid with a dual mechanism of action consisting of agonism at the mu opioid receptor and norepinephrine reuptake inhibition. Its development for use in children ages 2-7 years was through a multinational global clinical drug development program and is now available in Europe for the treatment of moderate to severe acute post operative pain [109]. It is reported to have an improved safety profile compared with tramadol in children as it has no analgesically active metabolite and is not significantly metabolized by cytochrome P450 enzymes [110]; however, it is currently not approved for any indication in children by the FDA.

Methadone acts as a mu-opioid receptor agonist and an NMDA receptor antagonist [111]. In addition, methadone also inhibits the reuptake of serotonin and norepinephrine [112]. It is a hydrochloride synthetic long-acting opioid with a phenyl heptylamine structure which makes it highly lipid-soluble [111]. The number of studies on its use in the pediatric population is limited. In the literature, methadone protocols have been published to prevent and treat iatrogenic opioid dependence in the pediatric population [111, 113] and manage post operative pain after major pediatric surgery [114, 115]. Methadone is routinely incorporated into pediatric cancer protocols for both neuropathic and nociceptive pain as well as end of life care [68, 69, 116-119].

#### 6.3.2 Opioid Considerations

It is important to recognize that, like other medication classes, there is a paucity of studies on the safety and efficacy of opioid use for pediatric neuropathic pain. A Cochrane review published in 2017 investigating the use of opioids in chronic, noncancer pain syndromes in children and adolescents could not identify any randomized trials that met their inclusion criteria [54]. Opioids should not be routinely considered for chronic use due to their adverse effects, abuse potential, tolerance, and physiologic dependence, but they may have a role in cancer related and intractable neuropathic pain management when non-pharmacologic approaches and non-opioid pharmacologic treatments fail [105]. A systematic approach to the initiation of opioid therapy must be outlined and documented, including 1) the use of the lowest effective dose, 2) a clear discussion about the goals of treatment and end points of therapy, 3) a risk screening for abuse potential and comorbid conditions, 4) the preferential use of short acting formulations, 5) documentation of opioid sparing strategies and/or prior failed pharmacologic and nonpharmacologic therapies, and 6) a review of all side effects, both common (constipation, nausea) and less common (significant cognitive impairment, opioid induced hyperalgesia) [105].

### 6.4 Non-steroidal Anti-inflammatories

There is a widespread belief that non-steroidal anti-inflammatories (NSAIDs) lack efficacy for the treatment of neuropathic pain. In fact, multiple neuropathic pain treatment guidelines do not mention this class of medication as potential adjuvant therapies [79, 80, 82]. Despite this, a study in 2004 in 55,686 adult patients with a variety of peripheral nerve disorders found that almost 40% of the cohort used NSAIDs compared with 14% of age-and sex-matched controls who did not have the same conditions. An topical review published in 2009 nicely highlighted this discrepancy and included relevant evidence supporting NSAID use for neuropathic pain in animal models [120]. A subsequent Cochrane review in adults found no evidence to support or refute the use of NSAIDs for neuropathic pain due to lack of data [121].

NSAIDs are similarly rarely discussed in treatment algorithms for neuropathic pain in children. The combination of NSAIDs and steroids was used successfully in a single case report for the treatment of recurrent painful ophthalmoplegic neuropathy (RPON), a rare neurological disorder predominantly seen in children, which is characterized by repeated headaches and reversible ipsilateral paresis of ocular cranial nerves [122]. A larger case series (n = 32) showed a marked response to indomethacin for a variety of headaches syndromes in children [123]. Beyond this, we were unable to find additional references to NSAID use in pediatrics for neuropathic pain.

### 6.5 Lidocaine

Lidocaine is an amino acid local anesthetic which prevents impulse initiation and transmission in the axons by blocking voltage dependent sodium channels [124]. When given intravenously, it acts on multiple voltage-gated channels as well as ligand gated calcium channels in the peripheral and central nervous system [125, 126]. In addition, preclinical data shows that lidocaine targets G-protein-coupled receptors and participates in cell signal transduction [125]. As continuous infusion, lidocaine produces an anti-allodynic effect [58] and has been shown to prolong pain interruption in rodent models of peripheral nerve injury [127].

#### 6.5.1 Intravenous Lidocaine

Clinically, lidocaine infusions have been used for a variety of acute and chronic pain indications including postoperative pain, neuropathic pain, visceral pain, hyperalgesia, and centrally mediated pain [124]. Research on lidocaine infusions to treat pain in adult populations has resulted in a large number of publications and associated systematic reviews [128, 129]. Specifically related to neuropathic pain, there have been multiple studies, including blinded randomized controlled trials, showing benefit of lidocaine infusions for improved pain relief without major adverse events [130-133]. The literature suggests that lidocaine infusions are generally well tolerated overall. Lightheadedness, nausea, vision changes, and lethargy are most reported [132].

In children, lidocaine infusions have been used in perioperative pain management, acute pain, chronic non-cancer pain, and cancer associated pain. A study published in 2014 reported the use of repeated, short lidocaine infusions (n = 58) in 15 pediatric patients with chronic, refractory pain conditions [134]. Of the included patients, 3 had known neuropathic conditions, 7 were identified as having mixed etiologies, and 5 had chronic headache. The type of chronic pain did not affect the analgesic efficacy with the mean pain reduction reported to be the same between groups. Patients described minimal side effects during the infusions with dizziness being the most common. In this study, lidocaine infusions were repeated every 4 weeks and run over 2-6 hours if the patient reported continued analgesic benefit. Another small case series reported 2 adolescents with sickle cell disease who received lidocaine infusions during 3 hospital admissions for pain associated with vaso-occlusive crisis with an opioid sparing effect [135]. In the pediatric oncology population, the use of lidocaine infusions has been reported for refractory pain [136, 137], treatment related pain [138-140], and neuropathic cancer pain [141]. Publications are limited to case reports, case series, and retrospective reviews.

Limitations of lidocaine infusions in clinical practice include the need for intravenous access, preferably with a dedicated lumen for safety and to ensure no issues with compatibility. While the pharmacokinetics of intravenous lidocaine in children have been described previously [142], there have been no studies evaluating dose response for neuropathic pain relief in pediatric patients. A study of 11 adults using computer controlled infusion pumps showed that when stable plasma lidocaine concentrations of 0.5, 1, 1.5, 2, and 2.5 micrograms/mL were targeted and maintained for 10 minutes, there was a significant plasma concentration-dependent decrease in pain scores starting at 1.5 micrograms/mL [143]. Finally, the use of lidocaine infusions is not without risk, particularly due to its narrow therapeutic window, and both clinical and laboratory monitoring of intravenous levels are important considerations to prevent Local Anesthetic Systemic Toxicity.

#### 6.5.2 Topical Lidocaine

Topical forms of lidocaine to treat localized neuropathic pain due to a variety of conditions have been reported as efficacious in adult studies [144-146]. Lidocaine 5% patches are not approved for use in the pediatric population, and no recommendations exist regarding their use in patients younger than 18 years [147]. Despite this, there have been 6 publications (n = 183) of analgesic and safety outcomes using 5% lidocaine medicated plasters for pediatric neuropathic pain conditions. The first reported use in children was published in a case series in 2008 of 5 patients who received lidocaine patches for neuropathic scar pain [148]. The authors report that the patches were effective in four of five patients, with complete resolution of pain in two of the patients at a 3-month follow up visit. Subsequent studies have used lidocaine patches in patients with neuropathic pain secondary to vaso-occlusive crisis who failed standardized treatment; a preliminary case series was published in 2013 (n = 6) [149] and a subsequent prospective multicenter clinical phase II trial was published in 2018 by the same authors (n = 40) [147]. The trial intervention was the application of a lidocaine plaster to the painful area for 12 hours per day for at least 3 consecutive days. 40 patients were enrolled across 4 pediatric centers and included patients with sickle cell vaso-occlusive crisis pain (n = 23), neuropathic pain (n = 11), and mixed neuropathic pain (n = 5) secondary to cancer. The primary outcome of the trial was a difference in the VAS pain score of at least 2 points between placement and removal of the patch over at least 2 out of the 3 days. The results of this study showed that the application of lidocaine patches over 3 consecutive days in children suffering from neuropathic pain or pain induced by sickle cell disease decreased the VAS score by at least 2 points in 59% of patients on day 1, 54% on day 2, and 67% on day 3.

An additional prospective study examined the effectiveness of 5% lidocaine patches and plasma lidocaine levels in 14 children with burn associated neuropathic pain over a 3-month period. All patients reported improved functionality and pain intensity and all plasma lidocaine levels were at least 180 times below critical levels. The largest study on the use of lidocaine 5% medicated plaster in children and adolescents was published in 2018 and included 115 patients with chronic peripheral and central neuropathic pain, chronic post-surgical and post-traumatic pain, and chronic primary pain [150]. Five centers participated in distributing self-report questionaries to eligible patients and collecting data. Results of this study showed that a total of 79 (69%) patients received benefit, 32 had no benefit, and data were incomplete for 4 patients. In the absence of randomized trials and guidelines for the treatment of localized neuropathic pain in children, the data from these studies further support the efficacy of the lidocaine 5% plaster in children and adolescents within the context of a multi-modal approach to pain management.

### 6.6 Other Pharmacologic Treatments

Other pharmacologic treatments may have a role in the management of neuropathic pain syndromes, but there is not enough evidence to support their routine use. Alpha2 agonists including clonidine, dexmedetomidine, and tizanidine may be adjuvant therapies in neuropathic pain syndromes through centrally mediated action on spinal interneurons. Clonidine and dexmedetomidine have been used in specific pediatric populations including children with severe neurological impairment [151] and during end of life palliative care for additional pain control [152]. Cannabinoids have been proposed as a treatment for spasticity, symptoms of multiple sclerosis (MS), and refractory neuropathic pain in adults; however, no studies have been performed on the use of medical marijuana in children. In 2015, the American Academy of Pediatrics published a policy statement opposing the use of cannabinoids outside of the regulatory FDA process but recognized it may be an option for children with refractory pain and life limiting conditions for which other current therapies are not adequate [153]. Carbamazepine and oxcarbazepine may be used in the treatment of trigeminal neuralgia; however, this condition is infrequently encountered in the pediatric population.

Ketamine use for neuropathic pain treatment in children is limited. Pediatric studies examining its use in neuropathic cancer pain and sickle cell vaso-occlusive crisis show opioid sparing effects [135, 141, 154], and it is included in published algorithms to manage pediatric cancer pain and palliative care [68, 116, 155]. A longitudinal study over 15 months in 63 in children with various etiologies chronic pain showed low dose ketamine infusions to be the most helpful in patients with CRPS, POTS, and a history of trauma-related chronic pain [156]. Contrary to the literature in adult patients, there are no studies of topical ketamine to treat pediatric neuropathic pain.

Topical capsaicin 8% patch is approved by the FDA for postherpetic neuralgia and diabetic neuropathy and by the EMA for all types of peripheral neuropathic pain. The mechanism of action is the activation of transient receptor potential cation channel subfamily V member 1 (TRPV1) ligand-gated channels on nociceptive fibers [31]. High concentration (8%) capsaicin results in an initial intense burning sensation and hypersensitivity of the skin following application. This period of increased sensitivity is followed by a period of reduced sensitivity and, after repeated applications, persistent desensitization [157]. In 2017, a Cochrane review which included 2488 adult participants from 8 studies found that topical high-concentration capsaicin used to treat postherpetic neuralgia, diabetic neuropathy and HIV neuropathy in adults was similar in its effects to other therapies from chronic neuropathic pain. A recent prospective study in 10 adolescent patients with sickle cell vaso-occlusive and chronic neuropathic pain was published in 2022 [158]. The primary outcome was the difference in pain threshold by mechanical QST. The topical capsaicin was generally well-tolerated and no serious side effects were observed. Pain thresholds at the treated site were significantly higher than that at an untreated site for seven of ten patients indicating that capsaicin may have a therapeutic benefit for localized neuropathic pain.

## 7 ONGOING THERAPEUTIC AND TECHNOLOGICAL INVESTIGATIONS

There are a number of emerging pharmacological approaches for the treatment of neuropathic pain [159]; however, it is unclear what role these agents will have for pediatric pain management. There are no active phase III clinical trials in children to treat neuropathic pain; therefore, this section will highlight Phase III clinical trials in adults and ongoing pre-clinical work. The oromucosal spray Nabiximols (trade name Sativex), a cannabis-based medication, has been evaluated in multiple phase III trials in adults with neuropathic pain, multiple sclerosis, diabetic neuropathy, and chemotherapy induced pain. This medication does not have FDA approval but has been approved in 30 countries, including the United Kingdom, Canada, and Germany, for the treatment of MS spasticity. Recent pre-clinical work has focused on the development of novel agents which inhibit neuroinflammation as well as repurposing drugs currently approved for other indications which have shown efficacy in animal models of neuropathic pain. Specifically, metformin [160] and simvastatin [161] have been identified as having antinociceptive and anti-inflammatory actions, particularly inhibiting the development of mechanical hyperalgesia by modulating ascending pain signaling pathways. There are ongoing investigations to identify and evaluate natural compounds with known anti-inflammatory properties and/or neuromodulation of pain pathways for the treatment of neuropathic pain [162, 163].

While new and repurposed pharmacological agents have not yet been investigated for neuropathic pain specifically in children, novel drug delivery systems using nanotechnology have been developed and present an opportunity to allow for more targeted treatments in pediatric populations [164]. Currently, nanoscience has been applied to cancer research; however, it is hypothesized that nanodrug delivery systems (NDDSs) have the potential to play a significant role in improving the effectiveness of current and future therapies for neuropathic pain. The emerging field of nanomedicine may be particularly relevant for application in children as a targeted approach at the molecular level that may prevent intolerable side effects, and NDDSs can be optimized in size, shape, and carrier which may provide pediatric patients more options for therapies.

## 8 PAIN PHENOTYPING: A MECHANISTICALLY DRIVEN APPROACH TO TREATMENT

The long-standing “trial and error” approach to the initiation of pharmacologic treatments for neuropathic pain in children and adolescents is not only not efficacious, but potentially harmful in the setting of medications with known substantial adverse side effects. Current published recommendations are either lacking entirely or not supported by evidence-based medicine. This comprehensive review shows the overall paucity of data available to make fundamental and nuanced practice decisions in the management of a challenging pain condition.

The current state requires a paradigm shift in the way pediatric pain is evaluated and managed. Recently published work on pain phenotyping using a combined QST/CPM based strategy has the potential to transform clinical treatment algorithms away from a diagnosis driven approach (*i.e*: neuropathic cancer pain *vs*. phantom limb pain) and towards precision medicine based on the manifestation of the underlying pain mechanism of the individual patient (Table **2**) [29].

In a recent study of 208 pediatric patients, tailoring treatment strategy by targeting pain phenotype was shown to reduce polypharmacy and interventional procedures without an increase in treatment failures [30]. Limitations to this approach include time needed to complete the QST/CPM testing, and protocols would likely require simplification in order to be incorporated into daily clinical practice. Nonetheless, developing treatment decisions based on the pain phenotype of the individual patient represents a promising way forward towards personalizing care and reducing harms in children and adolescents.

## CONCLUSION

The management of neuropathic pain is challenging, and there are unique considerations for its mechanisms, diagnosis, and treatment for children. Despite their widespread off-label use, there is little evidence to support pharmacologic agents for neuropathic pain, even those routinely proposed as first line therapies in the pediatric literature. Based on this review of the available data, our recommendations for pediatric patients with neuropathic pain are as follows: 1) Topical agents should be considered first line therapies if pain is localized. Considering their potential, we strongly encourage the research community to further study the efficacy and safety of topical medications in the pediatric population, especially considering the wide arrays of individual molecules and combinations used in adults that have yet to be investigated in the pediatric setting; 2) Whenever possible, sensory testing should be conducted to inform pharmacologic strategies and allow for an individualized, tailored approach to neuropathic pain management; 3) Given the known risks and unknown efficacy, the initiation of any systemic pharmacologic agent for neuropathic pain treatment in children must be carefully considered within the context of a broader interdisciplinary treatment plan.

## Figures and Tables

**Table 1:** Causes of neuropathic pain in children.

**Classification**	**Disease or Mechanism of Injury**	**Primary Location of Pain Mediation**
Cancer	Compression by tumor Nervous system invasion Chemotherapy induced	Peripheral Central Peripheral
Metabolic	Fabry’s Disease	Peripheral
Genetic	Erythromelalgia Charcot-Marie Tooth	Peripheral Peripheral
Trauma	Post-surgical Plexus avulsion Peripheral nerve transection Mechanical compression Burn-associated	Peripheral Peripheral and central Peripheral Peripheral Peripheral
Phantom-Limb	Amputation	Peripheral and central
Neurologic	Spinal Cord Injury Guillain-Barre Multiple Sclerosis	Central Peripheral Central

**Table 2:** Pharmaceutical treatment options depending on quantitative sensory testing/physical examination findings.

**QST/CPM Phenotype**	**Physical Examination Finding**	**Suggested Treatment: 1^st^ Line**	**Suggested Treatment: 2^nd^ Line**
Allodynia and/or temporal summation	Brush allodynia at site of pain	Gabapentinoids, Topical treatments	Ketamine (IV or oral), Opioids
Pain Pressure Sensitivity	Mechanical pressure hyperalgesia at the site of pain	NSAIDs, Topical treatments, Nerve blocks	Lidocaine IV infusions
Inefficient or Suboptimal Conditioned Pain Modulation	-	TCAs, Oral Clonidine	SNRIs or SSRIs

**Note**: Adapted from: Bruneau *et al*., Association between the Use of Quantitative Sensory Testing and Conditioned Pain Modulation and the Prescription of Medication and Interventional Procedures in Children with Chronic Pain Conditions, *Children* 2022, *9*, 1157-1170.

## LIST OF ABBREVIATIONS

CCI: Chronic Constriction Injury
CIPN: Chemotherapy Induced Peripheral Neuropathy
GABA: Gamma-aminobutyric Acid
MDD: Major Depressive Disorder
MID: Minimally Important Difference
MS: Multiple Sclerosis
NDDSs: Nanodrug Delivery Systems
NDRI: Norepinephrine Dopamine Reuptake Inhibitor
NSAIDs: Non-steroidal Anti-inflammatories
RCT: Randomized Controlled Trial
SNI: Spared Nerve Injury
SNRIs: Serotonin and Norepinephrine Reuptake Inhibitors
SSRIs: Selective Serotonin Reuptake Inhibitors
TCAs: Tricyclic Antidepressants
TLR: Toll Like Receptor
